# 

**Published:** 1890-10-18

**Authors:** 


[illegible]OVRIL the patients would in have gained strength the disease under suffering. I have no [illegible]scribe to your patients, to your convalescents, to those of [illegible]RIL, which, in a concentrated form, contains a substantial nine cases out of ten to battle against which they were hesitation to advise you your clients who have mental exertions, to use JOHNSTON'S tonic and a palatable food."—J. M. BEAUSOLIEL, M.D.

THROUGHOUT THE UNITED KINGDOM.

### THE HOSPITAL

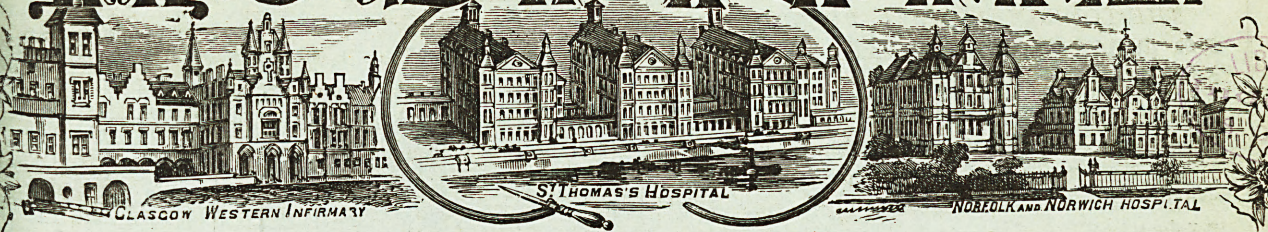

WITH EXTRA NURSING SUPPLEMENT.

No. 212. Vol IX.] SATURDAY, OCTOBER 18th, 1890. [ONE PENNY.

